# Combinational dual drug delivery system to enhance the care and treatment of gastric cancer patients

**DOI:** 10.1080/10717544.2020.1822460

**Published:** 2020-10-26

**Authors:** Ying Xiao, Yuewen Gao, Fajuan Li, Zhihe Deng

**Affiliations:** a Second Department of General Surgery, Xinxiang Central Hospital, Xinxiang, PR China; b Department of General Surgery, Rizhao People's Hospital, Rizhao, PR China; c Department of Gastroenterology, The First Affiliated Hospital of Guangdong Pharmaceutical University, Guangzhou, PR China

**Keywords:** Combinational drug delivery, gastric cancer, nursing care, apoptosis

## Abstract

Gastric cancer is a frequently occurring cancer with high mortality each year worldwide. Finding new and effective therapeutic strategy against human gastric cancer is still urgently required. Hence, we have established a new method to achieve treatment-actuated modifications in a tumor microenvironment by utilizing synergistic activity between two potential anticancer drugs. Dual drug delivery of gemcitabine (GEM) and Camptothecin-11 (CPT-11) exhibits a great anti-cancer potential, as GEM enhances the effect of CPT-11 treatment of human gastric cells by providing microenvironment stability. However, encapsulation of GEM and CPT-11 obsessed by poly(lactic-co-glycolic acid) (PLGA)-based nanoparticles (NPs) is incompetent owing to unsuitability between the binary free GEM and CPT-11 moieties and the polymeric system. Now, we display that CPT-11 can be prepared by hydrophobic covering of the drug centers with dioleoylphosphatidic acid (DOPA). The DOPA-covered CPT-11 can be co-encapsulated in PLGA NPs alongside GEM to stimulate excellent anticancer property. The occurrence of the CPT-11 suggestively enhanced the encapsulations of GEM into PLGA NPs (GEM-CPT-11 NPs). Formation of the nanocomposite (GEM-CPT-11 NPs) was confirmed by FTIR and X-ray spectroscopic techniques. Further, the morphology of GEM NPs, CPT-11 NPs, and GEM-CPT-11 NPs and NP size was examined by transmission electron microscopy (TEM), respectively. Furthermore, GEM-CPT-11 NPs induced significant apoptosis in human gastric NCI-N87 and SGC-791 cancer cells *in vitro*. The morphological observation and apoptosis were confirmed by the various biochemical assays (AO-EB, nuclear staining, and annexin V-FITC). In addition, evaluation of the hemolysis assay with erythrocytes of human shows excellent biocompatibility of free GEM, free CPT-11, GEM NPs, CPT-11 NPs, and GEM-CPT-11 NPs. The results suggest that GEM-CPT-11 NPs are one of the promising nursing cares for human gastric cancer therapeutic candidates worthy of further investigations.

## Graphical Abstract

**Figure:**
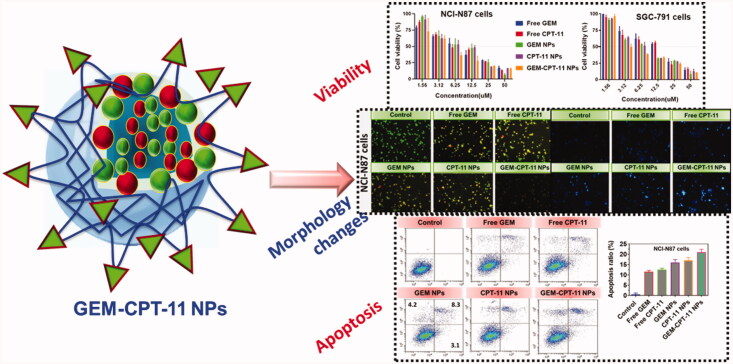

## 1. Introduction

Gastric cancer is the fourth most common cancer and the second most frequent cause of cancer-associated mortality worldwide. Although many treatment approaches, with maturing endoscopy therapy, chemotherapy, and surgery are used to treat gastric cancer, the results for persistent progressive gastric cancer are reduced (Yang et al., 2012; Lin et al., 2015; Zheng et al., 2019). The common patients unavoidably die from tumor recurrence or metastasis. Regrettably, till date, no active therapeutic approaches occur to solve this difficult. Hence, the progress of active antitumor agents to resist gastric cancer is a promising field (Wang et al., 2017; Liang & Yang, 2020; Rong et al., 2020). Also, the drug of selection for the action of gastric cancer, gemcitabine (GEM), suffers from poor extravasation into gastric cancer tissues and rapid enzymatic deamination upon circulation which produces its inactive metabolite, 2′,2′-difluorodeoxyuridine (dFdU) (Yan et al., 2019; Konstantinopoulos et al., 2020; Thompson et al., 2020). Moreover, the presence of a desmoplastic stromal around the cancer site creates a barrier for the drug. This results in high dosages of chemotherapy being required to attain an effect, which increases chances of side effects (Li et al., 2015). Thus, significant research efforts have been made toward the design of drug delivery systems targeted at improving the therapeutic outcomes of chemotherapy with GEM and Camptothecin-11 (CPT-11) (Meng et al., 2013; Sobot et al., 2016; Bernards et al., 2018; Jiang et al., 2019).

Combination therapy can be performed via co-administration of a supplementary cancer drug along with a sensitizer. The interfaces within potential anticancer drugs rely on the dose ratios between the two medications and can be potentially incompatible (Sasada et al., 2015; Bang et al., 2017; Jayanathan et al., 2020). Consequently, the importance of preserving a beneficial ratio to maintain a synergistic relationship between two drugs through nanoparticles (NPs) formulations cannot be ignored (Namiki et al., 2011; Wang et al., 2018; Zhu et al., 2020). The procedure of encapsulating several anticancer drugs in individual NPs has proved to be problematic because the drugs have to preserve their important physicochemical properties. Hence, nanoformulations that are prepared by encapsulating numerous medications with varied physico-chemical belongings while preserving controlled ratios are preferred for drug delivery within the body tissues (Yixuan et al., 2010; Li et al., 2012; Xin et al., 2013; Broza et al., 2018).

Nanoparticle-based drug delivery systems have been developed as a valuable system among other important methods for improved malignancy treatment (Ambrogio et al., 2013; Ge & Liu, 2013; Kumar et al., 2013; Florek et al., 2017). Appropriately, structured NPs can isolate the medications from the circulatory system and evade being eliminated by the renal system (Zhang et al., 2018; Li et al., 2019; Zhang et al., 2019, 2020). These NPs have an advanced system to deliver anticancer medications to targeted locations and decrease nonspecific harm to the target tissues, brought about through enhanced permeability and retention (EPR) effects (Zhou et al., 2014; Shen et al., 2016; Chen et al., 2020; Kumari et al., 2020; Martínez-López et al., 2020). Moreover, NP frameworks offer stable watery scattering of medications by surface adjustment and shield medications from degradation, resulting in improved anticancer action (Mirza & Karim, 2019; Ding et al., 2020; Zhou et al., 2020).

In this work, we have described a nanoplatform formed by encapsulation of two potential drugs into poly(lactic-co-glycolic acid) (PLGA) nanoparticles (GEM-CPT-11 NPs) via a nanoprecipitation method. Furthermore, *in vitro* cytotoxicity of the drug-loaded NPs was examined in human gastric cancer cells using an MTT assay. Additionally, we examined morphological changes in the treated cells by dual staining (AO-EB) and nuclear staining methods. Apoptosis was confirmed by the flow cytometry analysis.

## 2. Materials and methods

### 2.1. Materials

CPT-11 and GEM were purchased from TCI (Shanghai, China). Hydrolyzed polyvinyl alcohol (PVA, 85–90%, mol. wt. of 30–50 kDa) was obtained from TCI (Shanghai, China). PLGA polymers (monomer ratio 50:50; MW 7 kDa) were acquired from J&K (Shanghai, China).

### 2.2. Methods

#### 2.2.1. Encapsulation of GEM and CPT-11 in GEM-CPT-11 NPs

An oil/water solvent evaporation technique was adapted to encapsulation of CPT-11 and GEM in PLGA-NPs. Briefly, dioleoylphosphatidic acid (DOPA)-coated CPT-11 (50 µg) cores and GEM (50 µg) were added to a PLGA-NP solution in CHCl_3_ (100 mg in 350 µL). The emulsified 9% PVA was mixed into chloroformic solution in 3 mL PBS solutions. The emulsions were stirred for 24 h, and they evaporated the organic solvents. CPT-11- and GEM-loaded PLGA nanoparticles (GEM-CPT-11 NPs) were kept at −20 °C to be used for future studies.

A water/oil/water double emulsion solvent evaporation technique was used to fabricate the PLGA-NPs containing DOPA-coated CPT-11, GEM. Briefly, TMR-dextran (200 µL) was blended into a CPT-11 and GEM polymeric solutions in CHCl_3_ with sonications. These emulsions were consequently blended in a PVA-PBS solution, left for solvents evaporations (Gupta et al., 2017; Li et al., 2017; Safari et al., 2018; Guo et al., 2019). The emulsions were stirred for 24 h, and they evaporated the organic solvents.

### 2.3. Examination of *in vitro* drug release

Assessment of *in vitro* drug release kinetics was performed using a dialysis diffusion technique (Chourasiya et al., 2016; Stein et al., 2018; Hsu et al., 2020). GEM-CPT-11 NPs (3 mL), and CPT-11 and GEM (0.1 mg/mL equivalent concentration) solutions were placed into the end-wrapped dialysis covers. Next, they were retained into 20 mL of discharging medium comprising 0.2% Tween-80 in PBS pH 7.4. By stirring at 100 rpm on a detour shakers at 37 °C, the drug release medium was removed and an equivalent size of new medium was added. The drug-releasing profiles of CPT-11 and GEM were examined using an UV-vis spectrometer.

### 2.4. *In vitro* cytotoxicity

NCI-N87 and SGC-791 cells were obtained from the Cell Bank of Beijing (Beijing, China). The cells were maintained in RPMI 1640 culture and Dulbecco’s modified Eagle’s medium (DMEM) medium supplemented with 10% fetal bovine serum (FBS) and 100 mL^−1^ penicillin. Then, NCI-N87 and SGC-791 cells were incubated in a humid atmosphere with 5% CO_2_ at 37 °C. *In vitro* biochemical staining was obtained from Cell Signaling (Shanghai, China).

### 2.5. Apoptotic staining

The morphological changes of the NCI-N87 and SGC-791 cells were examined by biochemical staining, including acridine orange-ethidium bromide (AO-EB) and Hoechst 33344 staining. After incubating for 24 h, the cells were seeded at a concentration of 1 × 10^4^ onto 48-well plates. The cells were treated with free CPT-11, free GEM, CPT-11 NPs, GEM NPs, and GEM-CPT-11 NPs at 2.5 µM concentration for 24 h. On the following day, the staining solution was added. After incubating the plates with the staining solution, the plates were washed with PBS three times. Images were obtained using a fluorescence microscope (Accu Scope EXI-310) at a magnification of ×20 (Mohamed Subarkhan et al., 2016; Balaji et al., 2020; Deepika et al., 2020).

### 2.6. Flow cytometry/annexin V-PI staining

The flow cytometry examination was examined by using the Apoptosis Detection Kit of fluorescein isothiocyanate (FITC) (Cell Signaling, Shanghai, China) utilized to confirm the apoptotic ratio of NCI-N87 and SGC-791 cells. The cells were treated with free CPT-11, free GEM, CPT-11 NPs, GEM NPs, and GEM-CPT-11 NPs at 2.5 µM concentrations for 24 h. The cells were washed thrice by using trypsin, and suspended in 1× binding buffer (500 μL) with FITC Annexin V (5 μL) and of PI (10 μL). After 20 min incubation, the samples were analyzed by flow cytometry. The obtained results were investigated with the BD FACS CantoTM II flow cytometer (Yixuan et al., 2010; Subarkhan & Ramesh, 2016; Mohamed Subarkhan et al., 2018). 

### 2.7. Hemocompatibility assay

Human blood samples were obtained from the First Affiliated Hospital of Guangdong Pharmaceutical University. Red blood cells (RBCs) were obtained by centrifuging the samples at 1800 rpm for 5 min at 5 °C. The RBCs were washed with PBS three times and resuspended in 4 mL of PBS. Next, 0.1 mL of diluted RBCs was added to the free GEM, free CPT-11, GEM NPs, CPT-11 NPs, and GEM-CPT-11 NPs. in 0.5 mL PBS suspension at the corresponding concentrations and incubated for 4 h. After incubation, the samples were transferred onto 96-well plates. Hemolytic activity was determined by measuring at an absorbance of 570 nm. The control samples of the lyses buffer and 100% lyses buffer were also analyzed in these experimental procedures (Tramer et al., 2012; Evans et al., 2013; Liang et al., 2019; Mohamed Subarkhan et al., 2019). The proportion of hemolysis was determined as follows: % hemolysis=(As – An)/(Ap – An)×100%, where As denotes the absorbance of samples (free GEM, free CPT-11, GEM NPs, CPT-11 NPs, and GEM-CPT-11 NPs) at various concentrations (5, 10, 15, 20, and 25), and An and Ap denote the negative and positive controls, respectively.

## 3. Results and discussion

### 3.1. Structural morphology and characterization

**Figure 1.**
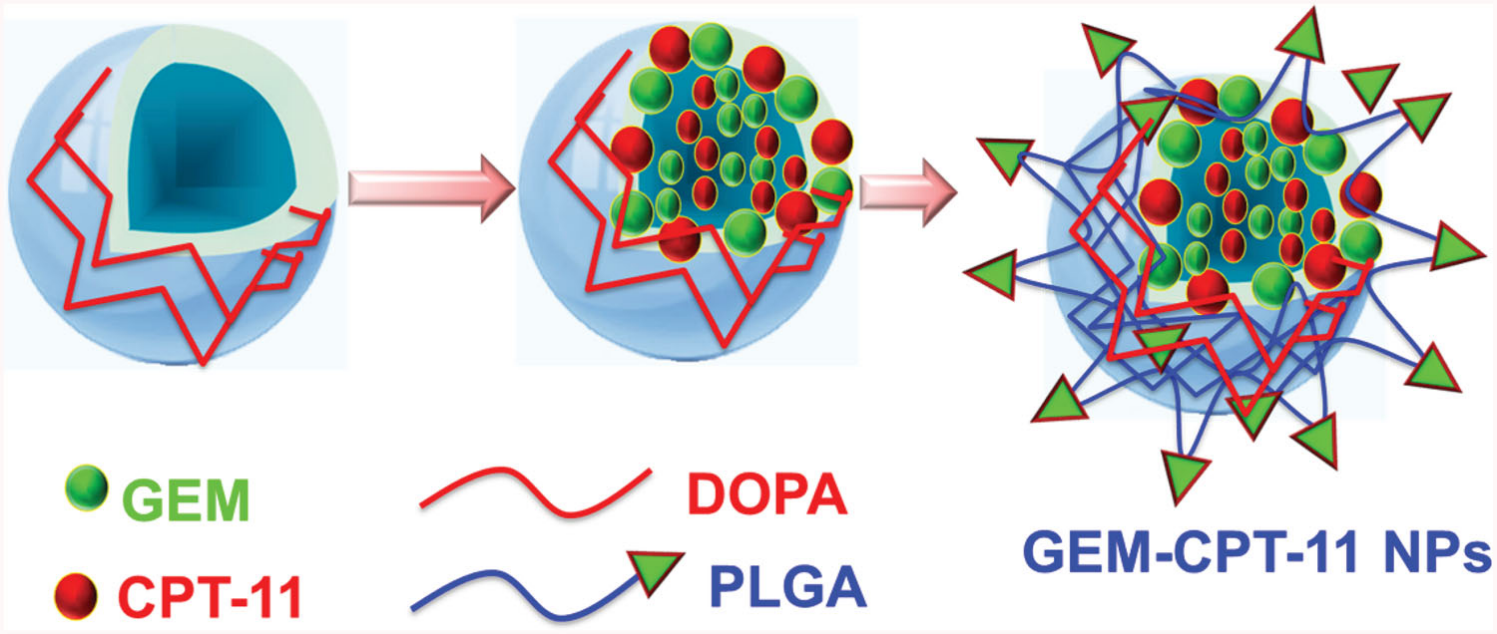
A schematic of the self-assembly of GEM and CPT-11 to form GEM-CPT-11 NPs of the treatment of gastric cancer.

Our achievement in proficiently stacking of CPT-11 (CPT-11) and GEM (GEM) into PLGA-NPs (designated as GEM-CPT-11 NPs) proposes another chance to co-deliver two medications for blend treatment. For instance, hydrophobic CPT-11 and GEM can be built into GEM-CPT-11 NPs simultaneously with other hydrophobic antitumor medications, such as GEM and paclitaxel. GEM was preferred for this study and its centers were embodied into GEM-CPT-11 NPs close to CPT-11, because of its cooperative energy with CPT-11. The main procedure of stacking of GEM and CPT-11 inside GEM-CPT-11 NPs is shown in Figure 1. GEM and CPT-11 are incorporated in the polymer framework of GEM-CPT-11 NPs done by hydrophobic interaction. Hence, the insertions are restricted by similarities concerning GEM and CPT-11 and their hydrophobic interaction with the co-polymer. Self-assembled nanoparticles (GEM-CPT-11 NPs) were formed spontaneously with 4 mg/mL CPT-11 and 8 mg/mL GEM by employing intermolecular hydrophobic interactions between the lipophilic moiety of GEM and CPT-11, as depicted in Figure 1.

**Figure 2.**
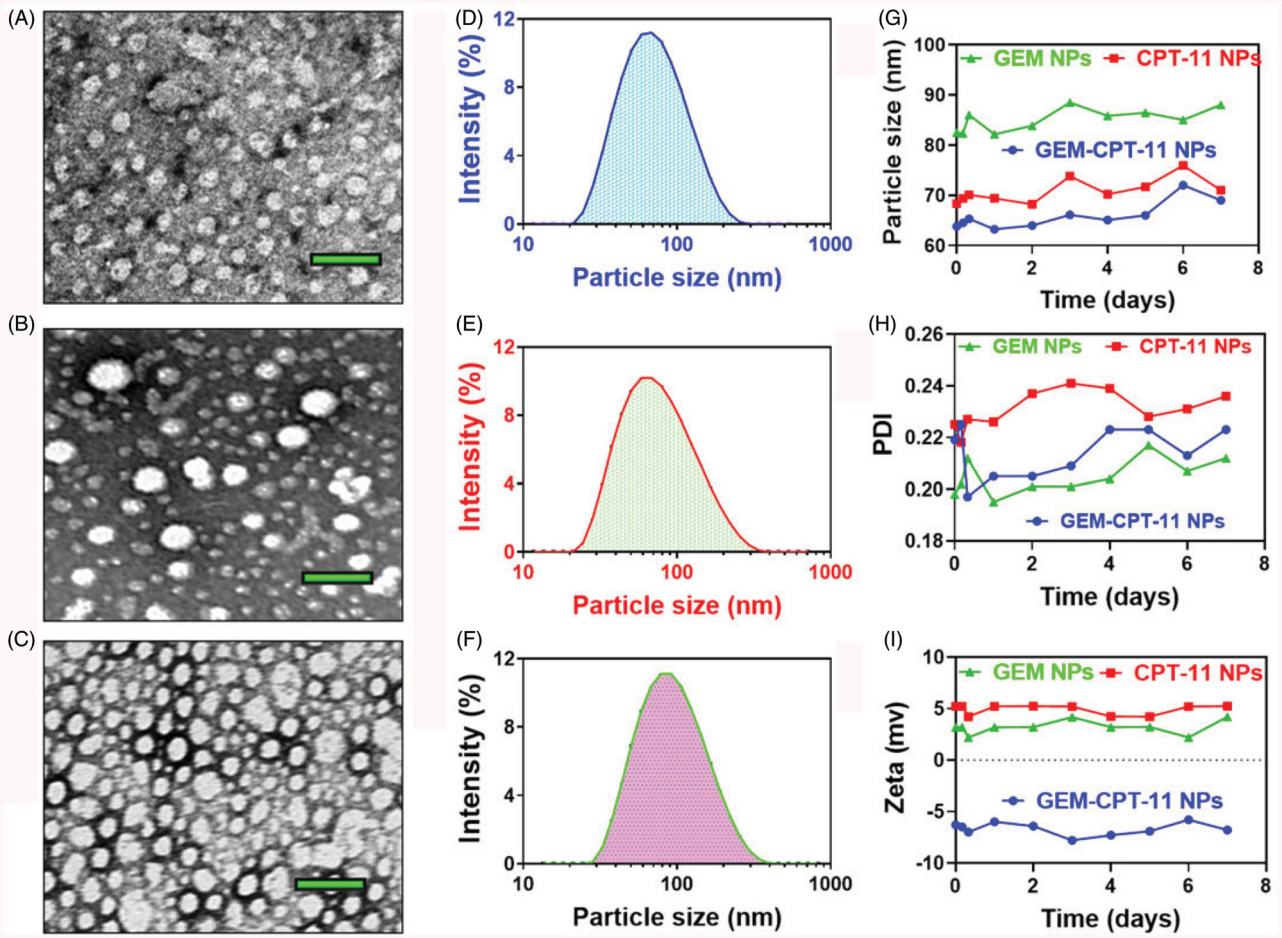
Characterization of the nanoparticles. (A–F) Morphology and particle size of GEM NPs, CPT-11 NPs, and GEM-CPT-11 NPs under a transmission electron microscope after negative staining with sodium phosphotungstate solution (2%, w/v). Scale bar: 20 nm. Particle size distribution of GEM NPs, CPT-11 NPs, and GEM-CPT-11 NPs analyzed by dynamic light scattering via a Zetasizer. (G–I) Stability of the GEM NPs, CPT-11 NPs, and GEM-CPT-11 examined by the dynamic light scattering.

The effects of the morphological surface of the hydrothermally prepared GEM NPs, CPT-11 NPs, and GEM-CPT-11 NPs were investigated through TEM analysis. The results as shown in Figure 2(A–C) depicts the creation of GEM-CPT-11 NPs. Additionally, morphological changes the synthesized polymeric NPs that were analyzed by HR-TEM. The nanocomposite was composed of agglomerated clusters of well-shaped hydroxyapatite nanocomposites (Figure 2(A–C)). The size of the GEM-CPT-11 NPs was examined by dynamic light scattering (DLS) analysis. The diameters of GEM NPs, CPT-11 NPs, and GEM-CPT-11 NPs measured from TEM images were in the range of 63.9 ± 0.3, 68.7 ± 0.5, 81.2 ± 0.9 nm (Figure 2(D–F)) and the polyplexes index was 0.277 ± 0.05, 0.252 ± 0.05, and 0.159 ± 0.02 for GEM NPs, CPT-11 NPs, and GEM-CPT-11 NPs, respectively, which is in agreement with the results of light scattering measurements and gives clear evidence of the size of the NPs compared to those analyses by TEM (Figure 2(D–F)). The stability of the GEM NPs, CPT-11 NPs, and GEM-CPT-11 NPs in PBS media was examined by determining the particle size of the GEM NPs, CPT-11 NPs, and GEM-CPT-11 NPs by DLS. Polyplexes index, specifically GEM NPs, CPT-11 NPs, and GEM-CPT-11 NPs, at an NPs ratio of 100:1 was organized and incubated for 30 min at 37 °C in order to confirm complete polyplex formation (Figure 2(G,H)). All the experiments were repeated three times. Additionally, the zeta potential and the stability of GEM NPs, CPT-11 NPs, and GEM-CPT-11 were determined to be 5.2 ± 0.4, 6.8 ± 0.5, and −6.3 ± 0.3 mV (Figure 2(I)) by DLS.

### 3.2. Controlled release of GEM-CPT-11 NPs

**Figure 3.**
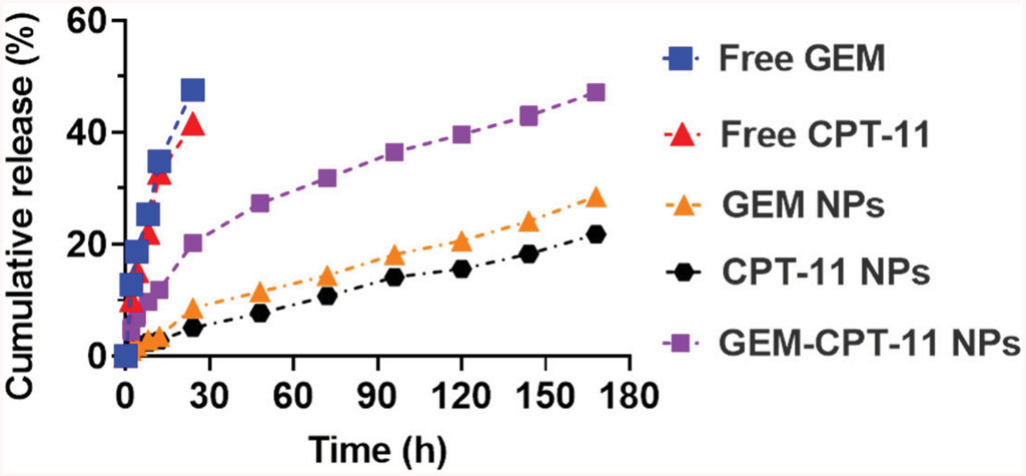
Drug release profiles (GEM and CPT-11) from the GEM NPs, CPT-11 NPs, and GEM-CPT-11 NPs against PBS containing 0.3% polysorbate 80.

Controlled release of GEM-CPT-11 NPs plays a vital role in the size, solubility, degradation, and drug loading by the NP frameworks. It is predictable that results confirm the drug release profile which shows the CPT-11 + GEM-loaded GEM-CPT-11 NPs reserve an enhanced efficiency to the frameworks. In contrast, if the drugs not deceived, a reckless and undesired untimely discharge will occur. These methods provide clues to the production of shell holes that permit the discharge of drugs. The controlled drug release was measured via physical and chemical analyses of the GEM-CPT-11 NPs and the encapsulation properties of the drugs. These dialysis methods were utilized to examine the outcomes of controlled release of the drugs encapsulated in the GEM-CPT-11 NPs and those associated with the free CPT-11 and GEM. The controlled release experiment was conducted in PBS at a pH of 7.2 at 37 °C. The controlled release profiles of the combination of CPT-11 and GEM loaded in the GEM-CPT-11 NPs displayed an initial release in about 5 h monitored via sluggish release for six days (Figure 3). First 10 h, half of the CPT-11 and GEM was discharged after the GEM-CPT-11 NPs formations. Subsequently, later 24 h, a gentle release of 40–50% was observed. These results indicate that the conjugation of CPT-11 and GEM on the surface of the PLGA-NPs (GEM-CPT-11 NPs) did not show any adverse effect on the controlled release by these nanocomposites.

### 3.3. *In vitro* cytotoxicity

**Figure 4.**
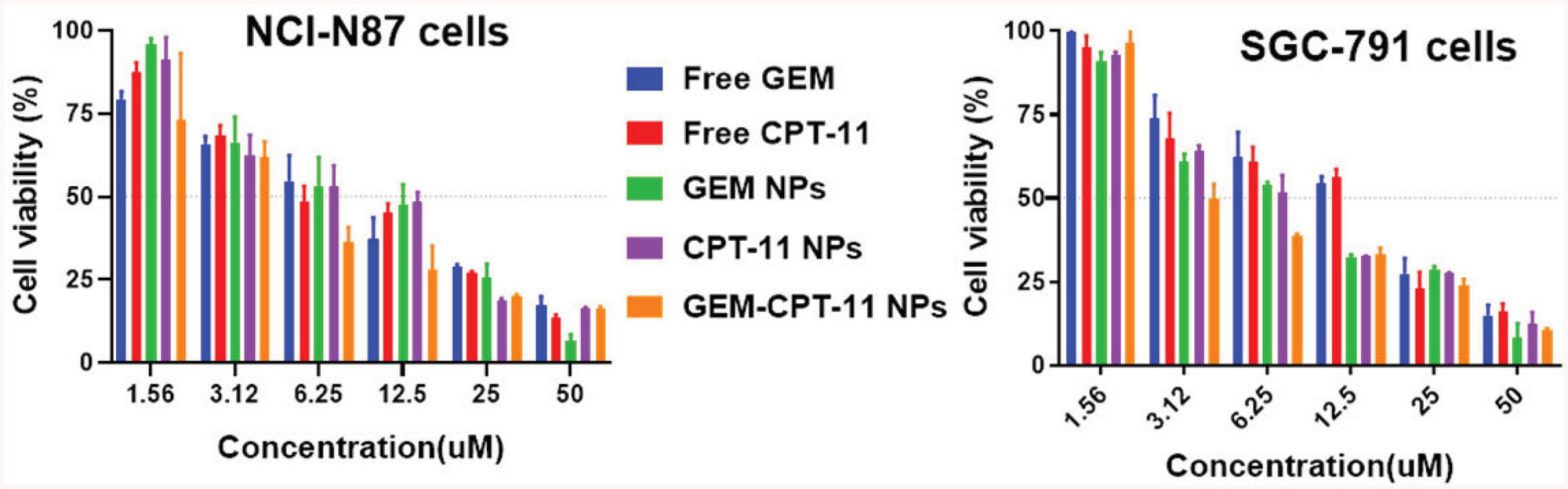
*In vitro* cytotoxicity of free CPT-11, free GEM, CPT-11 NPs, GEM NPs, and GEM-CPT-11 NPs were evaluated in NCI-N87 and SGC-791 gastric cancer cells. Cell viability was examined by the MTT assay after 24 h of drug incubation.

After successful synthesis of GEM-CPT-11 NPs, we performed an MTT assay to evaluate the cytotoxic effects of free CPT-11, free GEM, CPT-11 NPs, GEM NPs, and GEM-CPT-11 NPs on gastric cancer cell lines, comprising NCI-N87 and SGC-791 cancer cells. Following treatments with the medications for 24 h, the cell viability was monitored, and minimum-inhibitory concentrations (IC_50_) were obtained from the dose-dependent curve (Figure 4). Surprisingly, GEM-CPT-11 NPs displayed substantial improvement in cytotoxicity of the cancer cells. For instance, in NCI-N87 cell lines, IC_50_ of 10.91 ± 11.12, 10.35 ± 1.22, 9.05 ± 2.11, 9.46 ± 0.98, and 6.62 ± 0.97 was observed for free CPT-11, free GEM, CPT-11 NPs, GEM NPs, and GEM-CPT-11 NPs, respectively. In SGC-791 cell lines, IC_50_ of 19.27 ± 3.30, 17.70 ± 2.54, 11.20 ± 0.98, 10.22 ± 1.87, and 7.16 ± 2.80 for free CPT-11, free GEM, CPT-11 NPs, GEM NPs, and GEM-CPT-11 NPs was observed, respectively. The enhanced cytotoxicity of the GEM-CPT-11 NPs was owing to the entire release of the double potential anticancer medications into the tumor cells. The hydrophilic molecules of PLGA dispense the aqueous layer via a lipid bilayer for cell membrane penetration. Thus, the enhancement of cellular uptake requires the cell membrane nucleosides delivery for the proteins.

### 3.4. Morphological changes in NCI-N87 and SGC-791 human gastric cancer cells

**Figure 5.**
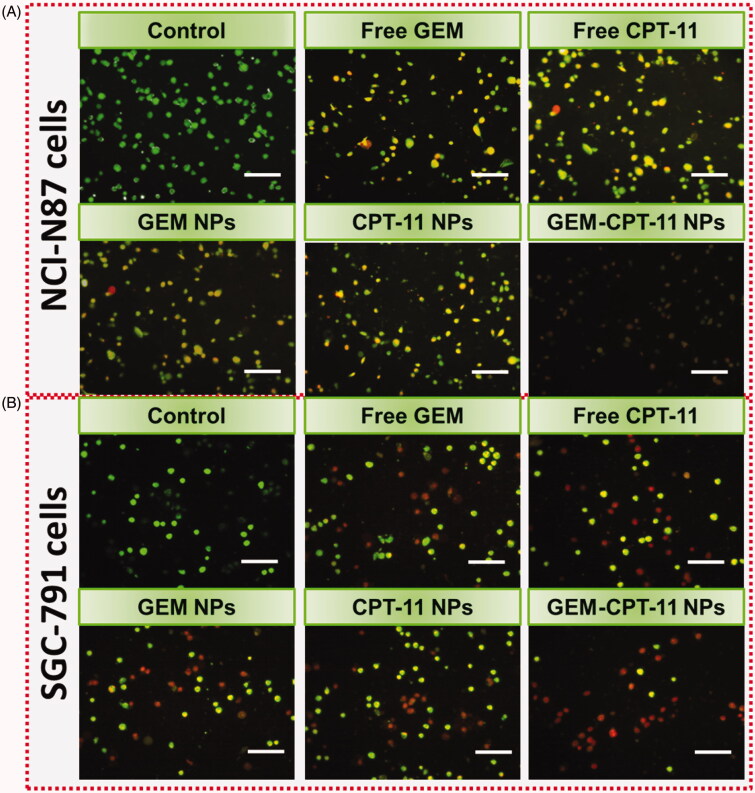
Dual AO/EB staining assay for examining free CPT-11, free GEM, CPT-11 NPs, GEM NPs, and GEM-CPT-11 NPs-induced cell death in NCI-N87 (A) and SGC-791 (B) cells. The cells were treated with free CPT-11, free GEM, CPT-11 NPs, GEM NPs, and GEM-CPT-11 NPs at 2.5 µM concentration for 24 h. Scale bar 20 µM.

Dual staining AO-EB is a qualitative technique used to identify live, early, late apoptotic, and necrotic cancer cells using fluorescent images to observe morphological changes in the nucleus of cells (Kasibhatla et al., 2006; Liu et al., 2015; Tambe et al., 2018). AO permeates the intacts membranes of usual and early apoptotic cell and binds to DNA, which fluoresces uniform green in normal cells and as patches in early apoptotic cells due to chromatin condensations. In difference, EB is only penetrable in the incapacitated membrane of late apoptotics and necrotics cell, where it fluoresces as bright orange patch through its bindings to DNA fragment or apoptotic moiety in late apoptotic cells, and as a unchanging orange fluorescence in the necrotic cell, as it has the nuclear changes in the morphology of viable cell. AO-EB-stained NCI-N87 and SGC-791 cells were incubated with free CPT-11, free GEM, CPT-11 NPs, GEM NPs, and GEM-CPT-11 NPs for 24 h. As presented in Figure 5, the presence of orange with reddish fluorescence with chromatin fragmentation after treatment of NCI-N87 and SGC-791 cells treated with free CPT-11, free GEM, CPT-11 NPs, GEM NPs, and GEM-CPT-11 NPs suggested that the GEM-CPT-11 NPs largely induced apoptosis in NCI-N87 and SGC-791 cells.

**Figure 6.**
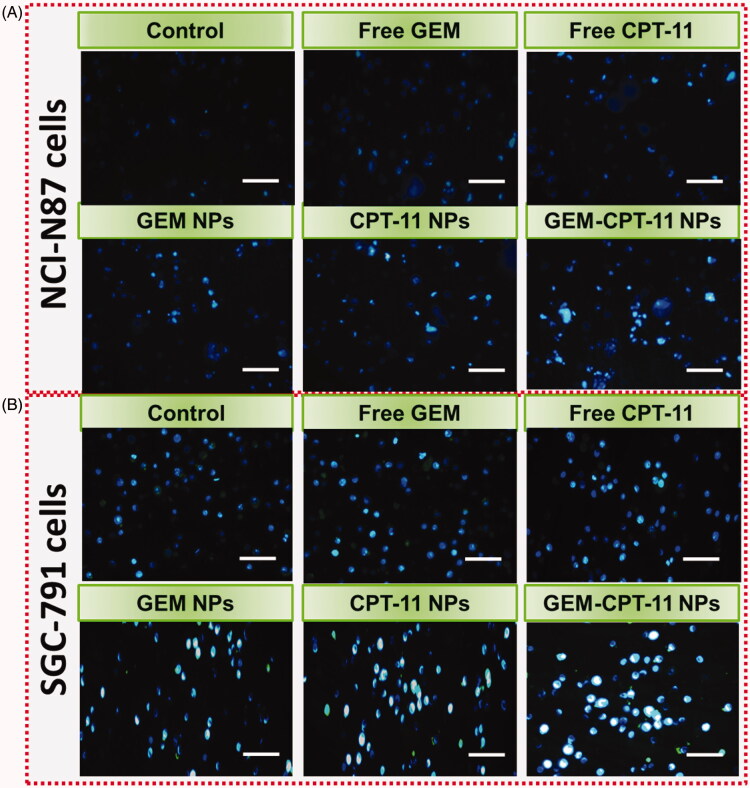
Nuclear (Hoechst 33258) staining assay for examining free CPT-11, free GEM, CPT-11 NPs, GEM NPs, and GEM-CPT-11 NPs-induced cell death in NCI-N87 (A) and SGC-791 (B) cells. The cells were treated with free CPT-11, free GEM, CPT-11 NPs, GEM NPs, and GEM-CPT-11 NPs at 2.5 µM concentration for 24 h. Scale bar 20 µM.

Hoechst 33258 staining was used to observe chromatin fragmentation, bi- and/or multinucleation, cytoplasmic vacuolation, nuclear swelling, cytoplasmic bleating, and late apoptosis in gastric cancer cells by visualizing dot-like chromatin condensation. Hoechst-33258-stained NCI-N87 and SGC-791 cells were incubated with free CPT-11, free GEM, CPT-11 NPs, GEM NPs, and GEM-CPT-11 NPs for 24 h. As displayed in Figure 6, the presence of blue fluorescence with chromatin condensation after treatment of NCI-N87 and SGC-791 cells treated with free CPT-11, free GEM, CPT-11 NPs, and GEM NPs suggested that the GEM-CPT-11 NPs largely induced apoptosis in NCI-N87 and SGC-791 (Figure 6).

### 3.5. Apoptosis in NCI-N87 and SGC-791 human gastric cancer cells

**Figure 7.**
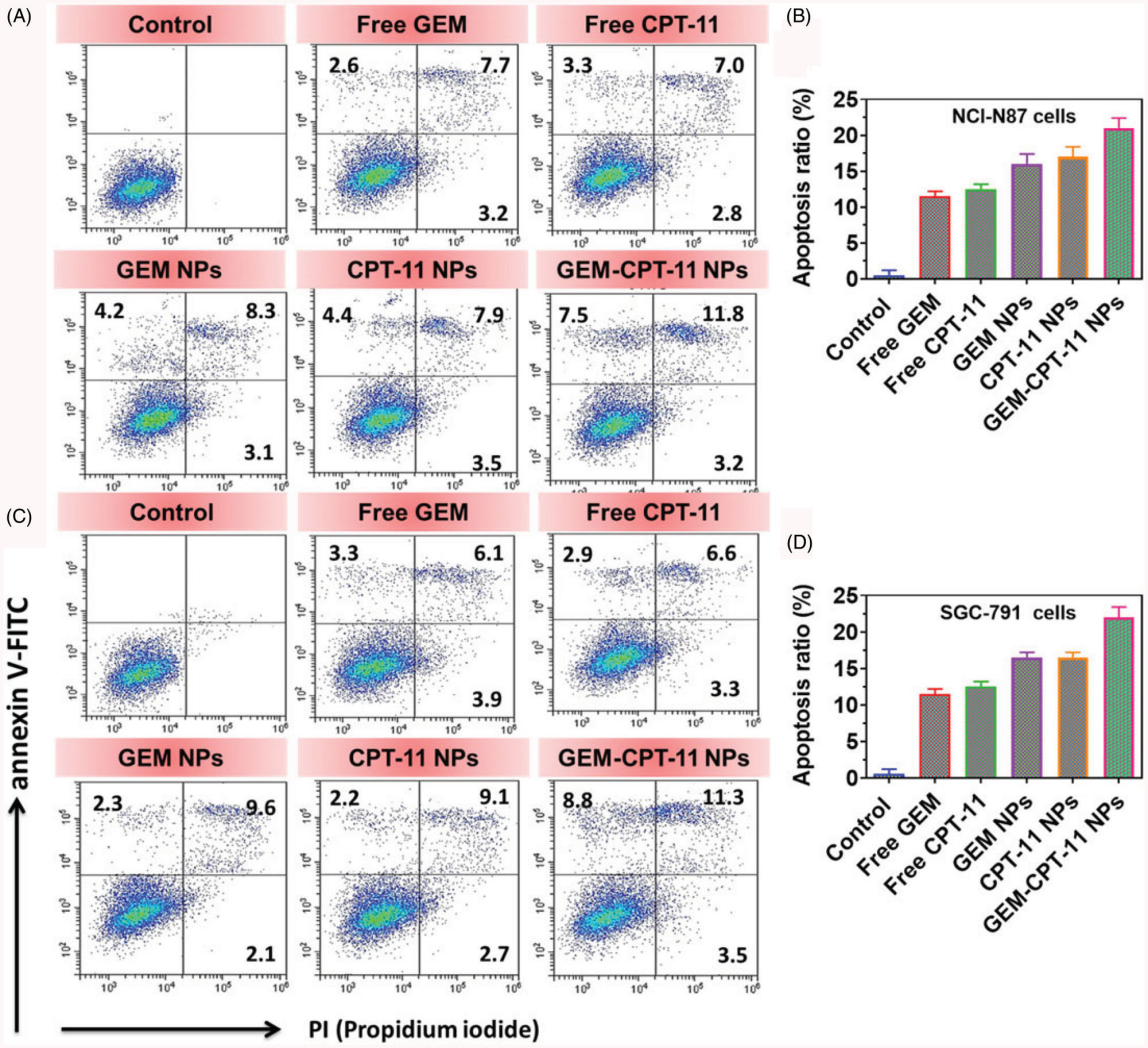
(A, C) Apoptotic analysis of NCI-N87 and SGC-791 cells using flow cytometry. The cells were treated with free CPT-11, free GEM, CPT-11 NPs, GEM NPs, and GEM-CPT-11 NPs at 2.5 µM concentration for 24 h and then stained with FITC annexin V/PI for flow cytometry analysis. (B, D) Apoptosis ratio of NCI-N87 and SGC-791 cells.

Apoptosis may be reckoned as an important obstacle for a damaged cell to become malignant tumors. Since the complexes promote apoptosis induction in cancer cells, flow cytometry using annexin V-FITC/propidium iodide (PI) double staining was carried out for the quantitative discrimination of apoptotic cells (Rehana et al., 2017; Mohan et al., 2018; Sathiya Kamatchi et al., 2020). Phosphatidylserine (PS) is a cell cycle signaling phospholipid located inner side of the membrane of a healthy cell but is reverted to the outer membrane for recognition by neighboring cells at the time of apoptosis. Hence, the translocation of PS is a morphological hallmark of apoptosis and can be spotted by its binding with fluorescently labeled annexin V which in turn detected by flow cytometry. Further, the addition of PI to annexin V stained cells is used to discriminate and concomitantly quantify the live cells (lower left quadrant-annexin V(–)/PI(–)), early apoptotic cells (upper left quadrant-annexin V(+)/PI(–)) and late apoptotic cells (upper right-quadrant-annexin V(+)/PI(+)) using FACS. As projected in Figure 7, the incubation of free CPT-11, free GEM, CPT-11 NPs, GEM NPs, and GEM-CPT-11 NPs with NCI-N87 and SGC-791 cells conspicuously induced apoptosis. It is worth to note that the titled complexes induce apoptosis even at very low concentrations which are less than their IC_50_. In comparison with control, the cell population was higher (6–9%) in annexin V(+)/PI(–) (upper left) quadrant indicating the induction of early apoptosis. This effect was ascertained to be high for GEM-CPT-11 NPs than the free CPT-11, free GEM, CPT-11 NPs, GEM NPs analogous with the results of MTT, and AO-EB staining assays. It is to note that the test samples displayed comparatively better apoptotic induction on NCI-N87 and SGC-791 cells.

### 3.6. Hemolysis assay in NCI-N87 and SGC-791 human gastric cancer cells

**Figure 8.**
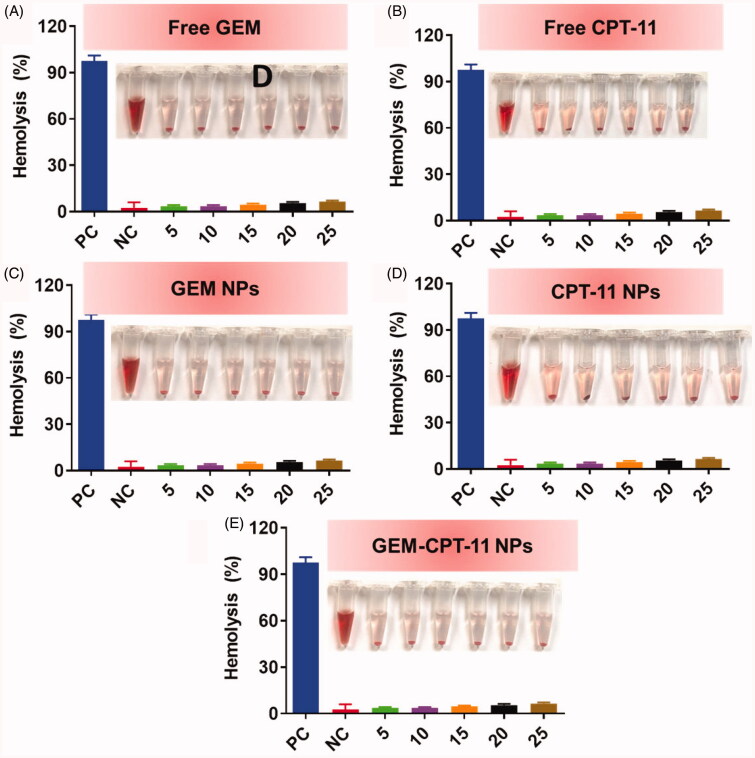
Biocompatibility of free CPT-11, free GEM, CPT-11 NPs, GEM NPs, and GEM-CPT-11 NPs with human blood.

The analysis of the interaction between NPs and human blood erythrocytes using hemolysis assays is the key in determining the blood compatibility of NPs (Figure 8). Free GEM, free CPT-11, GEM NPs, CPT-11 NPs, and GEM-CPT-11 NPs were found to display excellent biocompatibility with human RBCs, as shown in Figure 8. The role of the toxic substances appeared to be nano-specific. According to the IOS/Technical Report 7406, the hemolytic rate of NPs or materials is limited to 5%. The release of erythrocytes by Free GEM, free CPT-11, GEM NPs, CPT-11 NPs, and GEM-CPT-11 NPs was insignificant, indicating that the NPs had a negligible level of toxicity, and thus, they were safe to cells.

## 4. Conclusions

We developed GEM-CPT-11 NPs by encapsulating GEM and CPT-11 moieties to change the tumor microenvironment for improved drug accretion and additional anticancer activities. At first, CPT-11 was incorporated into GEM-CPT-11 NPs with effectual loading and encapsulation by direct self-assembly method. In this study, we showed that CPT-11 could be made hydrophobic by using an oil/water solvent evaporation method for drug delivery. These DOPA-covered CPT-11 centers were compatible with PLGA and could be co-encapsulated in GEM-CPT-11 NPs. The closeness of the CPT-11 centers fundamentally developed the epitome of GEM into PLGA-NPs. The formation of the nanocomposite was confirmed by FTIR and X-ray spectroscopic techniques. Further, TEM electroscopic techniques displayed the crystallized structure of the nanocomposite. GEM-CPT-11 NPs comprising double CPT-11 and GEM led to remarkable apoptosis in human gastric NCI-N87 and SGC-791 cancer cells. Further, morphological changes in the cells were monitored using dual staining and nuclear staining methods. AO-EB fluorescent staining and flow cytometry analysis reveal that all the complexes induce cancer cell death by apoptosis mechanism. Additionally, evaluation of the hemolysis assay with erythrocytes of human shows excellent biocompatibility of free GEM, free CPT-11, GEM NPs, CPT-11 NPs, and GEM-CPT-11 NPs. The preliminary results of the work established further investigation of the nursing cares *in vivo* examinations in future.
